# Validation of “Clinical Red Flags” for RA-ILD in an Italian cohort

**DOI:** 10.3389/fmed.2025.1625742

**Published:** 2025-07-16

**Authors:** Eneida Cela, Chiara Bonini, Francesco Cavalli, Alessandra Luciano, Barbara Kroegler, Josuel Ora, Alberto Bergamini, Marcello Chiocchi, Paola Rogliani, Maria Sole Chimenti, Paola Conigliaro

**Affiliations:** ^1^Rheumatology, Allergology and Clinical Immunology, Department of System Medicine, University of Rome “Tor Vergata”, Rome, Italy; ^2^Division of Respiratory Medicine, University Hospital Policlinico Tor Vergata, Rome, Italy; ^3^Department of Diagnostic Imaging and Interventional Radiology, University of Rome Tor Vergata, Rome, Italy

**Keywords:** interstitial lung disease, rheumatoid arthritis, dyspnea, cough, crackles

## Abstract

**Introduction:**

Rheumatoid arthritis (RA) is a systemic inflammatory disease, characterized by articular and extra-articular manifestations, including Interstitial Lung Disease (ILD). An early diagnosis of ILD can be essential in improving disease outcome. A clinical practice checklist has previously been proposed, highlighting red flags in signs and symptoms suggestive of RA-ILD. Our aim was to validate the “checklists of red flags signs or symptoms suggestive of RA-ILD” in our cohort of RA patients, by assessing the diagnostic utility of dyspnea, cough, and crackles, both individually and in combination.

**Methods:**

We performed a retrospective study including medical charts of consecutive RA patients fulfilling 2010 ACR/EULAR classification criteria. The diagnosis of RA-ILD was based on the chest HRCT exam. The primary symptoms and signs of ILD, namely cough, crackles, and dyspnea, were considered separately and in combination to determine diagnostic performance metrics.

**Results:**

Our cohort included 107 patients with RA, from which 55 (51.4%) with a diagnosis of RA-ILD. Female patients were predominant in both RA-ILD and No-ILD groups (56.4 and 82.6% respectively), with a significantly higher proportion in the latter (*p* = 0.0036). Dyspnea alone demonstrated a good diagnostic utility for RA-ILD with a sensitivity of 63.5% and specificity of 60%, PPV of 60% and an NPV of 63.5%, (*p* = 0.0203). Additionally, crackles exhibited the highest sensitivity among the individual symptoms (66.7%), a specificity of 57.4% and a significant association with RA-ILD (*p* = 0.0265). The presence of either dyspnea or crackles confirmed their strong association with RA-ILD (*p* = 0.0066), with the highest level of accuracy (63.5%) and specificity (63.8%). Also, the combination of cough or dyspnea was significantly associated with RA-ILD (*p* = 0.0111). A strong correlation was observed between RA-ILD and the presence of both crackles and dyspnea (*p* = 0.0351). When the three symptoms were combined, the sensitivity was 64.3%, the specificity was 53.2%, the PPV was 32.7%, and the NPV was 81%, but did not reach statistical significance (*p* = 0.1284).

**Conclusion:**

Overall, crackles and dyspnea were the most significant markers of RA-ILD, both individually, and in combination. This study confirms that the red flags previously identified, especially in combination, show an important accuracy and reliability as clinical biomarkers in the early detection of RA-ILD.

## Introduction

Rheumatoid arthritis (RA) is a chronic inflammatory disease that manifests mainly with erosive symmetric polyarthritis (1). The estimated prevalence is 0.5–1% (2, 3). Besides synovial involvement, RA is a systemic disorder characterized by extra-articular manifestations, including Interstitial Lung Disease (ILD) (4, 5). ILD is considered to have a prevalence ranging from 5 to 58%, depending on the diagnostic tools used (5, 6). Early diagnosis of ILD can be difficult, as pulmonary involvement may firstly be subclinical in about 11.9–55.7% of patients (7, 8). Generally, RA-ILD presents with dyspnea, dry cough, chest discomfort, and asthenia. At clinical examination, patients with RA-ILD can present bilateral basal crackles in almost 90% of cases (9, 10). Typically, the onset of these manifestations occurs within 5 years of RA diagnosis, but may precede joint involvement in 20% of patients (11, 12). Previous studies have shown that male gender, seropositivity, bone erosions, and cutaneous nodules are risk factors for RA-ILD (13–16).

In the early stages of RA-ILD, up to 64% of patients may present with a normal chest radiograph, making conventional X-rays insufficient for early detection (17, 18). Therefore, High-Resolution Computed Tomography (HRCT) of the chest remains the gold standard for identifying interstitial lung involvement in RA.

The earliest abnormalities detectable on HRCT include subpleural reticulations and ground-glass opacities, which typically reflect inflammatory activity. Over time, these findings may evolve into more specific fibrotic patterns. Among them, usual interstitial pneumonia (UIP) is the most common radiological pattern associated with RA-ILD. Less frequently observed patterns include nonspecific interstitial pneumonia (NSIP) and organizing pneumonia (OP) (19, 20).

UIP is characterized by a subpleural and basal predominance of reticular abnormalities, honeycombing, traction bronchiectasis, and architectural distortion—findings that reflect disease chronicity and severity (21). UIP has been associated with a more aggressive clinical course, a higher risk of progression, and a poorer response to treatment compared to NSIP (22).

NSIP, although less prevalent, typically shows a combination of ground-glass opacities, reticular changes, and lower lobe volume loss with subpleural sparing. It exists in both fibrotic and cellular forms, with the former being more common in RA-ILD. OP is the least frequent pattern and is characterized by focal consolidations, ground-glass opacities, and the reversed halo sign (18).

In RA patients, the presence of interstitial lung abnormalities on HRCT has significant prognostic and therapeutic implications. Given the potential for rapid progression and the high impact on morbidity and mortality, early radiological identification is crucial. For this reason, a multidisciplinary approach involving rheumatologists, pulmonologists, and radiologists is recommended to enhance diagnostic accuracy and optimize patient management (23, 24).

## Materials and methods

We performed a retrospective study including medical charts of consecutive RA patients, evaluated in an outpatient clinic from 1st May 2022 to 30th April 2024. The inclusion criteria were:

1. Adults aged ≥ 18 years.
2. Diagnosis of RA according to the 2010 American College of Rheumatology/European League Against Rheumatism (ACR/EULAR) classification criteria and performed at least 6 months before.
3. The presence of a thoracic HRCT, performed during the follow up.

Exclusion criteria:

1. Missing data during follow up.
2. Patients with a history of other autoimmune diseases that may affect the lungs, such as Systemic Sclerosis, Sjögren’s syndrome, Mixed Connective Tissue Disease, Undifferentiated Connective Tissue Disease or Idiopathic Inflammatory Myositis.
3. Active bacterial, viral or mycotic infections of the respiratory tract at the time of radiological evaluation.

The diagnosis of RA-ILD was based on the chest HRCT exam and was performed through a blind evaluation by two radiology (M.C. and A.L.) and two pneumology specialists (J.O. and F.C.).

The primary symptoms and signs of ILD, namely cough, crackles, and dyspnea, were considered separately and in combination to determine diagnostic performance metrics such as sensitivity, specificity, positive predictive value (PPV), negative predictive value (NPV) and overall accuracy, calculated based on 2 × 2 contingency tables. The association between each symptom and RA-ILD was assessed using the Chi-square test and Fisher’s exact test to determine statistical significance (*p* < 0.05). Additionally, a multivariate analysis was conducted using binary logistic regression to evaluate the independent associations between each red flag symptom (dyspnea, crackles, and cough) and known risk factors for RA-ILD. The independent variables included sex (male), smoking status (ever vs. never smoker), rheumatoid factor (RF) positivity, anti-citrullinated protein antibodies (ACPA) positivity, and Disease Activity Score 28 (DAS28). Each symptom was used as a binary dependent variable (presence vs. absence), and odds ratios (ORs) with 95% confidence intervals (CIs) were calculated. A *p*-value < 0.05 was considered statistically significant. Statistical analyses were performed using GraphPad Prism 8.0.1 (GraphPad Software Inc., San Diego, CA, United States) and SPSS ver. 24 (IBM Corp., Armonk, NY, United States). The study protocol was approved by ethic committee of the Policlinico Tor Vergata, Rome.

## Results

Our cohort included 107 medical records of patients with RA, of whom 55 (51.4%) had a diagnosis of RA-ILD, according to chest HRCT scans (Table 1). Female patients were predominant in both RA-ILD and No-ILD groups (56.4 and 82.6% respectively), with a significantly higher proportion in the latter (*p* = 0.0036). No significant differences were observed in terms of the mean age at RA diagnosis. The mean age at the ILD diagnosis was 67.4 ± 10.4 years, and in these patients, we observed a significantly longer disease duration (*p* < 0.0001) and a higher prevalence of rheumatoid factor (RF) (*p* = 0.0036), anti-citrullinated protein antibodies (ACPA) (*p* = 0.0049), and both (*p* = 0.0077). The multivariate logistic regression analysis did not reveal any statistically significant associations between the presence of individual red flag symptoms (dyspnea, crackles, and cough) and known risk factors for RA-ILD, including male sex, smoking history, RF positivity, ACPA positivity, and elevated DAS28 scores. Bronchiectasis was significantly prevalent in the RA-ILD than in the No-ILD group (41.8% vs. 11.5% respectively, *p* = 0.0005). Therapies were similar across both groups, except for a significantly higher use of Abatacept in RA-ILD patients (*p* = 0.0006). Table 2 shows the sensitivity, specificity, PPV, NPV, accuracy and *p*-value of the red flags. Dyspnea alone demonstrated a good diagnostic utility for RA-ILD. It showed a sensitivity of 63.5% and specificity of 60%, with a PPV of 60% and a NPV of 63.5%, highlighting a significant association with RA-ILD (*p* = 0.0203).

**Table 1:** Demographic and clinical characteristics of the cohort.

Variables	RA	RA-NoILD	RA-NILD	*p* value
*N*	107	55	52	
Female, *n* (%)	74 (69.1)	31 (54.6%)	43 (82.6)	**0.0036**
Age at RA diagnosis (mean ± SD)	64.9 ± 12.8	65 0.4 ± 14.1	64.5 ± 11.7	0.2084
RA disease duration (mean ± SD)	9.05 ± 6.85	9.8 ± 8.1	8.27 ± 5.18	**<0.0001**
Age at ILD diagnosis (mean ± SD)	/	67.4 ± 10.4	/	
Positive Rheumatoid factor, *n* (%)	85 (79.4)	50 (90.9)	35 (67.3)	**0.0036**
Positive ACPA, *n* (%)	83 (77.6)	49 (89.1)	34 (65.4)	**0.0049**
Double positivity for FR and ACPA	79 (73.8)	47 (85.5)	32 (61.5)	**0.0077**
Smoking, *n* (%)	54 (50.5)	30 (54.5)	24 (46.2)	0.4415
Bronchiectasis	29 (27.1)	23 (41.8)	6 (11.5)	**0.0005**
Dyspnea	52 (48.6)	33 (60.0)	19 (36.5)	**0.0203**
Crackles	39 (36.4)	26 (47.3)	13 (25.0)	**0.0265**
Cough	52 (48.6)	30 (54.5)	22 (42.3)	0.2474
Non productive	27 (25.2)	21 (38.2)	6 (11.5)	**0.0018**
Productive	25 (23.4)	9 (16.4)	16 (30.8)	0.1094
Therapy				
csDMARDs	97 (90.6)	49 (89.1)	48 (92.3)	0.7427
MTX	90 (84.2)	44 (80.0)	46 (88.5)	0.2939
LEF	37 (34.6)	20 (36.4)	17 (32.7)	0.8391
bDMARDs	72 (67.3)	37 (67.3)	35 (67.3)	>0.9999
TNFα-i	58 (54.2)	25 (45.5)	33 (63.3)	0.081
ABA	31 (29.0)	24 (43.6)	7 (13.7)	**0.0006**
IL6-i	18 (16.8)	9 (16.4)	9 (17.9)	>0.9999
RTX	13 (12.1)	9 (16.4)	4 (7.4)	0.2386
JAK-i	5 (4.7)	3 (5.5)	2 (3.2)	>0.9999
Antifibrotic therapy	3 (2.8)	3 (5.45)	/	

SD, Standard Deviation; ILD, Interstitial Lung Disease; RA, Rheumatoid Arthritis; RA-ILD, Rheumatoid Arthritis – associated Interstitial Lung Disease; RA-NoILD, Rheumatoid Arthritis – without Interstitial Lung Disease; ACPA, Anti-Citrullinated Peptide Antibodies; RF, Rheumatoid Factor; csDMARDs, conventional synthetic Disease-modifying antirheumatic drugs; MTX, Methotrexate, LEF, Leflunomide; bDMARDs, biological Disease-modifying antirheumatic drugs; TNFαi, Tumor Necrosis Factor alpha inhibitor; ABA, Abatacept, IL6-i, Interleukin 6 inhibitor; RTX, Rituximab; JAK-i, Janus Kinase Inhibitor. Significant *p* values are indicated in bold.

**Table 2:** Diagnostic reliability of clinical red flags for RA-ILD.

Variables	Sensitivity	Specificity	PPV	NPV	Accuracy	*p*-value
Dyspnea, (%)	63.5	60	60	63.5	61.7	**0.0203**
Cough, (%)	57.7	54.6	54.6	57.7	56.1	0.2474
Crackles, (%)	66.7	57.4	47.3	75	60.1	**0.0265**
Dyspnea or cough, (%)	62	63.6	71	53.4	59.6	**0.0111**
Dyspnea or crackles, (%)	63.3	63.8	69.1	57.7	63.5	**0.0066**
Cough or crackles, (%)	57.1	55	58.1	53.9	56.1	0.2481
Dyspnea and cough, (%)	58.5	53	43.6	67.3	55.1	0.3202
Dyspnea and crackles, (%)	67.7	55.3	38.2	81	58.9	**0.0351**
Cough and crackles, (%)	64.5	54	36.4	79	57	0.092
Dyspnea and cough and crackles, (%)	64.3	53.2	32.7	81	56.1	0.1284

Significant *p* values are indicated in bold.

Additionally, crackles exhibited the highest sensitivity among the individual signs, with a sensitivity of 66.7%, a specificity of 57.4% and a significant association with RA-ILD (*p* = 0.0265). The PPV was 47.3%, and the NPV was 75%.

On the other hand, cough demonstrated the lowest diagnostic utility among the symptoms evaluated.

As a second step we considered these signs and symptoms also in combinations, including the presence of at least one of the red flags or the identification of two or more items at the same time.

The presence of either dyspnea or crackles confirmed their strong association with RA-ILD even in the combined analysis, with the highest level of accuracy (63.5%), specificity (63.8%) and of statistical significance (*p* = 0.0066).

The combination of cough or dyspnea was also evaluated, showing a strong association with RA-ILD (*p* = 0.0111). Cough or crackles (*p* = 0.2481) and cough with dyspnea together (*p* = 0.3202), on the other hand, did not significantly correlate with RA-ILD, indicating limited diagnostic value.

A strong correlation was observed between RA-ILD and the presence of both crackles and dyspnea (*p* = 0.0351). The cough and crackles combination also demonstrated some potential diagnostic utility, however, the *p*-value of 0.0927 indicates a trend toward significance rather than clear-cut diagnostic relevance. When the three symptoms and signs—cough, crackles, and dyspnea—were combined, the sensitivity was 64.3%, the specificity was 53.2%, the PPV was 32.7%, and the NPV was 81%, but did not reach statistical significance (*p* = 0.1284).

## Discussion

Rheumatoid arthritis-associated interstitial lung disease (RA-ILD) is a serious complication that may significantly impact the prognosis and the outcome of RA patients. Considering the high morbidity and mortality rate of ILD among RA patients, a prompt detection and treatment are crucial.

Smoking is a known risk factor for the development of RA, especially by promoting the development of ACPAs (15, 25). It has been observed a correlation between smoking and RA-ILD (17), although a few studies did not find an association, possibly due to a threshold effect for smoking on RA-ILD risk (26). On the other hand, Idiopathic Pulmonary Fibrosis (IPF) shows a strong and confirmed correlation with a history of smoking (27). In our cohort, although we did not observe a significant difference in smoking history between RA patients with or without ILD (*p* = 0.4415), RA-ILD patients presented significantly higher rates of RF and ACPA (*p* = 0.0036 and *p* = 0.0049, respectively). This suggests that while smoking may partly contribute to disease pathogenesis, additional pathways and environmental or genetic factors likely coexist and modulate ILD development in RA.

Previously demographic, serologic and genetic features were evaluated as potential risk factors for RA-ILD development, useful in the screening of RA patients for pulmonary involvement (11, 16, 28). Johnson et al. (29) identified: high-titer rheumatoid factor, high-titer anti-CCP, cigarette smoking, older age at rheumatoid arthritis onset, high disease activity, male sex, higher body mass index as risk factors for developing RA-ILD. In our study we did not observe a significant correlation between these disease features and the presence of dyspnea, cough or crackles, although RF and ACPA positivity were significantly related to RA-ILD development. Other biomarkers have been identified as related to ILD such as anti-carbamylated proteins (Anti-CarP), Krebs von den Lungen 6/MUC1 (KL-6), as well as a genetic background including predisposing genes such as HLADRB1*15, HLADRB1*16, DQB1*06, and HLA-A*31:01, or single nucleotide variations of MUC5B gene (11, 24). While these elements may provide an important insight, clinical manifestations reported by the patient or evidenced during the examination could furtherly and more precisely direct toward the presence of an eventual RA-ILD. Hackner et al., proposed a Delphi-based screening strategy for the detection and the follow up of RA-ILD including serological and clinical features. Nevertheless, this risk factor algorithm was not singularly weighted or confirmed on real life cohort studies. Bosello et al. (30) through a multidisciplinary consensus panel, elaborated a checklist of clinical signs including dyspnea, cough and crackles, that could have an impact in the early detection of RA-ILD. The aim of our study was to validate these red flags in a real-world clinical practice, by measuring their degree of diagnostic accuracy in a monocentric cohort of RA patients.

Our results show that both dyspnea and crackles are extremely important predictors of RA-ILD on their own and collectively. Dyspnea alone, exhibited a modest diagnostic utility, with sensitivity of 63.5% and specificity of 60%, with PPV of 60%. Most importantly, dyspnea demonstrated a statistically significant relationship with RA-ILD. These findings are in accordance with other studies that highlighted dyspnea as an early and frequent sign of RA-ILD; reflecting its role as a red flag symptom in clinical practice (31–33). It is noteworthy that the moderate specificity indicates overlap with other possible pulmonary issues, hence, further pulmonary functional and imaging examinations will be needed for confirmation.

Out of the three red flags, crackles resulted the most sensitive (66.7%), highlighting their valuable clinical impact in the detection of RA-ILD. These results align with prior research indicating that bibasal crackles may be detected in nearly 90% of RA-ILD patients (11). The strong negative predictive value of 75% could indicate that the absence of crackles may rule out RA-ILD, although a more comprehensive evaluation is needed, considering the subclinical cases of the disease (8).

On the other hand, cough showed lower diagnostic utility as a stand-alone symptom, consistently with previous findings in literature that indicated it as a common, although less specific and reliable symptom in the RA-ILD evaluation (34).

The analysis of combined red -flags highlighted that the presence of either dyspnea or crackles yielded the highest diagnostic accuracy (63.5%) and specificity (63.8%), along with a statistically significant association with RA-ILD. Furthermore, the simultaneous presence of both dyspnea and crackles, appeared to be a robust indicator significantly related to RA-ILD. Similarly, the cough or dyspnea combination demonstrated a significant correlation with RA-ILD confirming the concept that combining multiple signs and symptom in the clinical evaluation could enhance diagnostic precision.

All three symptoms and signs combined—dyspnea, cough, and crackles—produced modest diagnostic metrics and did not reach statistical significance suggesting that while this pairing may have potential, it requires further exploration in larger cohorts.

To date, chest HRCT is the gold standard for the diagnosis of RA-ILD (17, 18). Chest ultrasound (US) has emerged as a potentially valuable tool for screening purposes in patients with rheumatoid arthritis. Chest US is widely available, non-invasive, and free from radiation exposure (35). Recent studies have demonstrated its high sensitivity and strong negative predictive value in detecting interstitial involvement, suggesting that chest US could serve as a complementary method to clinical evaluation in the screening of patients to furtherly evaluate through HRCT (35, 36).

This study highlights the complexity of RA-ILD disease and its early detection, underscoring the necessity of a comprehensive clinical, serologic and radiologic evaluation, alongside with a multidisciplinary approach.

This study encompasses some limitations such as the limited cohort, the monocentric and the retrospective validation analysis of this red flags. Therefore, further research in larger, multicentric and longitudinal cohorts would be important to assess and evaluate the impact of these red flags and monitor their evolution with disease progression.

## Conclusion

Overall, crackles and dyspnea were the most significant markers of RA-ILD, both individually, and in combination. Our results confirmed that dyspnea, with a high sensitivity and a strong predictive value, serves as a red flag symptom, warranting further investigation for RA-ILD. Additionally, the presence of crackles is a valuable clinical sign useful as a diagnostic marker for RA-ILD, particularly when considering its high sensitivity and strong NPV. This study confirms that the red flags previously identified, especially in combination, show an important accuracy and reliability as clinical biomarkers in the early detection of RA-ILD.

## Data Availability

The raw data supporting the conclusions of this article will be made available by the authors, without undue reservation.

## References

[1] Smolen JS Aletaha D McInnes IB. Rheumatoid arthritis. Lancet. (2016) 388:2023–38. doi: 10.1016/S0140-6736(16)30173-8 27156434

[2] Bernardinello N Zen M Castelli G Cocconcelli E Balestro E Borie R. Navigating interstitial lung disease associated with rheumatoid arthritis (RA-ILD): from genetics to clinical landscape. Front Med. (2025) 12:1542400. doi: 10.3389/fmed.2025.1542400 PMC11891064 40066169

[3] Gabriel SE. The epidemiology of rheumatoid arthritis. Rheum Dis Clin North Am. (2001) 27:269–81. doi: 10.1016/s0889-857x(05)70201-5 11396092

[4] De Benedittis G Latini A Morgante C Bonini C Cela E Kroegler B. Alteration of telomere length and mtDNA copy number in interstitial lung disease associated with rheumatoid arthritis. Autoimmunity. (2025) 58:741. doi: 10.1080/08916934.2025.2473741, PMID: 40035723

[5] Atzeni F Siragusano C Gómez-Puerta JA. Interstitial lung disease and rheumatoid arthritis. Handbk Syst Autoimmune Dis. (2022) 17:21–40. doi: 10.1016/B978-0-323-91083-5.00010-4

[6] Román Ivorra JA Trallero-Araguas E Lopez Lasanta M Cebrián L Lojo L López-Muñíz B. Prevalence and clinical characteristics of patients with rheumatoid arthritis with interstitial lung disease using unstructured healthcare data and machine learning. RMD Open. (2024) 10:e003353. doi: 10.1136/rmdopen-2023-003353 38296310 PMC10836356

[7] Conigliaro P Triggianese P De Martino E Fonti GL Chimenti MS Sunzini F. Challenges in the treatment of rheumatoid arthritis. Autoimmun Rev. (2019) 18:706–13. doi: 10.1016/j.autrev.2019.05.007 31059844

[8] Hackner K Hütter L Flick H Grohs M Kastrati K Kiener H. Screening for rheumatoid arthritis-associated interstitial lung disease—a Delphi-based consensus statement. Z Rheumatol. (2024) 83:160–8. doi: 10.1007/s00393-023-01464-w PMC10902070 38240817

[9] Fazeli MS Khaychuk V Wittstock K Han X Crocket G Lin M. Rheumatoid arthritis-associated interstitial lung disease: epidemiology, risk/prognostic factors, and treatment landscape. Clin Exp Rheumatol. (2021) 39:1108–18. doi: 10.55563/clinexprheumatol/h9tc57 33635222

[10] Joy GM Arbiv OA Wong CK Lok SD Adderley NA Dobosz KM. Prevalence, imaging patterns and risk factors of interstitial lung disease in connective tissue disease: a systematic review and meta-analysis. Eur Respir Rev. (2023) 32:220210. doi: 10.1183/16000617.0210-2022 36889782 PMC10032591

[11] Florescu A Gherghina FL Mușetescu AE Pădureanu V Roșu A Florescu MM. Novel biomarkers, diagnostic and therapeutic approach in rheumatoid arthritis interstitial lung disease—a narrative review. Biomedicine. (2022) 10:1367. doi: 10.3390/biomedicines10061367, PMID: 35740390 PMC9219939

[12] Hyldgaard C Hilberg O Pedersen AB Ulrichsen SP Løkke A Bendstrup E. A population-based cohort study of rheumatoid arthritis-associated interstitial lung disease: comorbidity and mortality. Ann Rheum Dis. (2017) 76:1700–6. doi: 10.1136/annrheumdis-2017-211138 28611082

[13] Huang S Doyle TJ Hammer MM Suzanne C Huang W Marshall AA. Rheumatoid arthritis-related lung disease detected on clinical chest computed tomography imaging: prevalence, risk factors, and impact on mortality. Semin Arthritis Rheum. (2021) 50:1216–25. doi: 10.1016/j.semarthrit.2020.08.015 PMC7736489 33059295

[14] Zhang Y Li H Wu N Dong X Zheng Y. Retrospective study of the clinical characteristics and risk factors of rheumatoid arthritis-associated interstitial lung disease. Clin Rheumatol. (2017) 36:817–23. doi: 10.1007/s10067-017-3561-5 28191607

[15] Correia CS Briones MR Guo R Ostrowski RA. Elevated anti-cyclic citrullinated peptide antibody titer is associated with increased risk for interstitial lung disease. Clin Rheumatol. (2019) 38:1201–6. doi: 10.1007/s10067-018-04421-0, PMID: 30645754 PMC8166218

[16] Wang H-F Wang Y-Y Li Z-Y He P-J Liu S Li Q-S. The prevalence and risk factors of rheumatoid arthritis-associated interstitial lung disease: a systematic review and meta-analysis. Ann Med. (2024) 56:40. doi: 10.1080/07853890.2024.2332406 PMC10984230 38547537

[17] Kelly CA Saravanan V Nisar M Arthanari S Woodhead FA Price-Forbes AN. Rheumatoid arthritis-related interstitial lung disease: associations, prognostic factors and physiological and radiological characteristics--a large multicentre UK study. Rheumatology. (2014) 53:1676–82. doi: 10.1093/rheumatology/keu165 24758887

[18] Zamora-Legoff JA Krause ML Crowson CS Ryu JH Matteson EL. Patterns of interstitial lung disease and mortality in rheumatoid arthritis. Rheumatology. (2016) 56:kew391. doi: 10.1093/rheumatology/kew391 PMC6251572 27940586

[19] Kim EJ Collard HR King TE. Rheumatoid arthritis-associated interstitial lung disease. Chest. (2009) 136:1397–405. doi: 10.1378/chest.09-0444 19892679 PMC2818853

[20] Yunt ZX Chung JH Hobbs S Fernandez-Perez ER Olson AL Huie TJ. High resolution computed tomography pattern of usual interstitial pneumonia in rheumatoid arthritis-associated interstitial lung disease: relationship to survival. Respir Med. (2017) 126:100–4. doi: 10.1016/j.rmed.2017.03.027 28427540

[21] Salaffi F Carotti M Di Carlo M Tardella M Giovagnoni A. High-resolution computed tomography of the lung in patients with rheumatoid arthritis. Medicine (Baltimore). (2019) 98:e17088. doi: 10.1097/MD.0000000000017088 31567944 PMC6756733

[22] Lucchino B Di Paolo M Gioia C Vomero M Diacinti D Mollica C. Identification of subclinical lung involvement in ACPA-positive subjects through functional assessment and serum biomarkers. Int J Mol Sci. (2020) 21:162. doi: 10.3390/ijms21145162, PMID: 32708286 PMC7404103

[23] Sebastiani M Vacchi C Cassone G Atzeni F Biggioggero M Carriero A. THU0150 interstitial lung disease related to rheumatoid arthritis. What do we don’t know? The LIRA study (lung involvement in rheumatoid arthritis). Ann Rheum Dis. (2020) 79:290–1. doi: 10.1136/annrheumdis-2020-eular.3516

[24] Johnson C. Recent advances in the pathogenesis, prediction, and management of rheumatoid arthritis-associated interstitial lung disease. Curr Opin Rheumatol. (2017) 29:254–9. doi: 10.1097/BOR.0000000000000380 28207496

[25] Klareskog L Malmström V Lundberg K Padyukov L Alfredsson L. Smoking, citrullination and genetic variability in the immunopathogenesis of rheumatoid arthritis. Semin Immunol. (2011) 23:92–8. doi: 10.1016/j.smim.2011.01.014 21376627

[26] Koduri G Norton S Young A Cox N Davies P Devlin J. Interstitial lung disease has a poor prognosis in rheumatoid arthritis: results from an inception cohort. Rheumatology. (2010) 49:1483–9. doi: 10.1093/rheumatology/keq035 20223814

[27] Cerri S Spagnolo P Luppi F Richeldi L. Smoking-related interstitial lung disease. Orphan Lung Dis. (2011) 54:282–300. doi: 10.1183/1025448x.10008710

[28] Narváez J Aburto M Seoane-Mato D Bonilla G Acosta O Candelas G. Screening criteria for interstitial lung disease associated to rheumatoid arthritis: expert proposal based on Delphi methodology. Reumatol Clin. (2023) 19:74–81. doi: 10.1016/j.reumae.2021.12.003 35753951

[29] Johnson SR Bernstein EJ Bolster MB Chung JH Danoff SK George MD. Guideline for the screening and monitoring of interstitial lung disease in people with systemic autoimmune rheumatic diseases. Arthritis Rheumatol. (2024) 76:1201–13. doi: 10.1002/art.42860 38973714 PMC12646464

[30] Bosello SL Beretta L Del Papa N Harari S Palmucci S Pesci A. Interstitial lung disease associated with autoimmune rheumatic diseases: checklists for clinical practice. Front Med. (2021) 8:761. doi: 10.3389/fmed.2021.732761 PMC8554062 34722574

[31] Swigris JJ Danoff S Dellaripa PF Doyle TJ Solomon JJ. Reliability and responsiveness of the D12 and validity of its scores as a measure of dyspnoea severity in patients with rheumatoid arthritis-related interstitial lung disease. BMJ Open Respir Res. (2023) 10:e001872. doi: 10.1136/bmjresp-2023-001872 PMC10533789 37748807

[32] Swigris JJ Yorke J Sprunger DB Swearingen C Pincus T du Bois RM. Assessing dyspnea and its impact on patients with connective tissue disease-related interstitial lung disease. Respir Med. (2010) 104:1350–5. doi: 10.1016/j.rmed.2010.03.027 20471238 PMC2914213

[33] Manfredi A Cassone G Cerri S Venerito V Fedele AL Trevisani M. Diagnostic accuracy of a velcro sound detector (VECTOR) for interstitial lung disease in rheumatoid arthritis patients: the inspireate validation study (INterStitial pneumonia in rheumatoid ArThritis with an electronic device). BMC Pulm Med. (2019) 19:111. doi: 10.1186/s12890-019-0875-x 31221137 PMC6587236

[34] Vermant M Kalkanis A Jacob J Goos T Cortesi EE Cypers H. Lung ultrasound outperforms symptom-based screening to detect interstitial lung disease associated with rheumatoid arthritis. RMD Open. (2025) 11:e005283. doi: 10.1136/rmdopen-2024-005283, PMID: 40010942 PMC12083264

[35] Ottaviani S Debray M-P Borie R Forien M Ebstein E Juge P-A. Value of lung ultrasonography for screening interstitial lung disease in patients with rheumatoid arthritis. Rheumatology. (2025) 4:keaf133. doi: 10.1093/rheumatology/keaf133 40037792

[36] Otaola M Vasarmidi E Ottaviani S Gutierrez M Dalpiaz MS Gaser A. Performance of lung ultrasound as a screening tool for subclinical rheumatoid arthritis-associated interstitial lung disease. Chest. (2025) 167:1687–95. doi: 10.1016/j.chest.2024.11.038 39675519

